# Teaching Reading: A Case Study Through Mixed Methods

**DOI:** 10.3389/fpsyg.2020.01083

**Published:** 2020-06-10

**Authors:** Natalia Suárez, Juan E. Jiménez, Carmen R. Sánchez

**Affiliations:** ^1^Departamento de Didácticas Específicas, Universidad de la Laguna, San Cristóbal de la Laguna, Spain; ^2^Departamento de Psicología Evolutiva y de la Educación, Universidad de la Laguna, San Cristóbal de La Laguna, Spain; ^3^Departamento de Psicología Clínica, Psicobiología y Metodología, Universidad de La Laguna, San Cristóbal de La Laguna, Spain

**Keywords:** beliefs, teaching practices, reading, teacher discourse, triangulation, mixed methods

## Abstract

The present study analyzes the relationship between teachers’ beliefs about learning to read, teaching practices, and discourse. To carry out this study, we benefited from the collaboration of six teachers in kindergarten and the first levels of primary education. First, an attribution questionnaire was used to analyze beliefs about learning to read (Jiménez et al., 2015). Secondly, to study teaching practices, an observation tool was used (Suárez et al., 2018). Thirdly, in order to know the opinion of teachers about how to teach reading, we adapted the instrument to assess teaching perspectives elaborated by Clark and Yinger (1979). Finally, all the information was triangulated and analyzed using mixed methods. The results indicated that the relationship between beliefs, practices, and discourse is not always consistent. In all teachers, a relationship was found between some of their beliefs, practices, and discourse. At the level of beliefs, all teachers presented one predominant attributional profile, although to a lesser extent, their beliefs were also attributable to other learning theories. The results indicated that all the teachers carried out teaching practices associated with the different learning theories. Similarly to their discourse, all teachers showed diverse opinions about the learning processes involved in reading. These results indicate that teachers maintain eclectic approaches, both when they carry out activities in the classroom and when they think about learning to read.

## Introduction

For almost three decades, research has documented the influence of teachers’ beliefs on educational practice (Berthelsen and Brownlee, 2007; Kuzborska, 2011; Barrot, 2015). Teacher’s beliefs are thoughts, perceptions, and values about their roles as educators, education, and how students learn (Vartuli, 2005). It has even been shown that if teachers are aware of their own beliefs, the repertoire of teaching skills can be increased (Tracey and Mandel, 2012), leading to a change in classroom decision making, and teaching strategies and evaluation. If we want to achieve improvements in teaching, it is necessary to examine the teachers’ beliefs and modify them (McAlpine and Weston, 2002). A great deal of research in this direction has shown that instructional events can be catalysts for changing beliefs (Stevens, 2002; Theurer, 2002; Fazio, 2003), since beliefs are permeable mental structures susceptible to change (Thompson, 1992), although there appears to be no consensus on this (Block and Hazelin, 1995; Richardson, 1996).

More recent studies have provided us with more detailed information on how beliefs and implicit knowledge influence teachers’ instructional practices (Cunningham and Zibulsky, 2009), actions, and strategies that they implement to teach reading in the classroom. The research carried out in this regard has focused on differentiating three traits appearing in the teaching and learning of reading. Thus, Tolchinsky and Ríos (2009) analyzed the relationship between what teachers say and do (2.250), teaching practice (*N* = 2), and students’ knowledge (*N* = 814). To do this, they used a self-report questionnaire of 30 questions, with high reliability (α = 0.81) and a Likert scale (0–6). Through a cluster analysis, they detected three differentiated profiles: *instructional* practices focused on teaching the names of letters, letter–sound relationships, as well as the importance of learning products; a *situational* approach to activities arising from classroom situations, where students look for the means to understand texts that they do not know; and *multidimensional* activities such as letter knowledge, recognition, and letter–sound association, as well as reading and writing work from situations that arise in the classroom. The results showed the following distribution: instructional (33.87%), situational (37.06%), and multidimensional (29.06%). Also, they found that 30% of the children were able to recognize unknown words and did not seem to have difficulty in mastering the code, and that teachers used explicit, early, and systematic teaching practices.

Also, in Spain, Barragán and Medina (2008), analyzed the practices teachers use through questionnaires. They found significant differences depending on the profile and educational level. Thus, nursery/kindergarten teachers showed a higher profile of situational practices (50%), compared to elementary school teachers who showed a profile of instructional practices (70%). Subsequently, they analyzed the profile of practices according to geographical area, finding that the teachers who carried out the greatest number of situational practices were those of the Basque country, followed by teachers from Almería, Cantabria, Catalonia, and the Community of Madrid (more than 50%). Catalonia and Cantabria showed a lower frequency of instructional practices (less than 20%); however, the teachers from León and Asturias used these practices more frequently (more than 55%). The same authors also observed six Early Childhood Education classrooms in Almeria. The results showed a relationship between the declared belief profile and its practices in the classroom. In another study, Ríos et al. (2010) demonstrated the relationship between the knowledge learned and the practices in teaching reading of two Infant Education teachers. They found that the contents worked on by the teacher with a situational profile were reading and writing functions, identification of words in reading, and letter names and sound values.

The teacher with an instructional profile used word identification and word reading. In the study carried out by Baccus (2004), a direct relationship was found between the teachers’ beliefs and the instructional time dedicated to the teaching of reading. In addition, Rapoport et al. (2016) focused on analyzing the beliefs that teachers maintain (*N* = 144) regarding the contribution of executive functions in reading performance and their teaching practice. Their results showed a positive relationship between these two variables (*r* = 0.512, *p* < 0.01).

Ethnicity has been another feature highlighted in studies assessing the dyad of beliefs and practices in teaching. The Center for the Improvement of Early Reading Achievement [CIERA] (2001) examined the beliefs and practices of 250 early childhood teachers. Their results showed a relationship between beliefs (based on the importance of the development of alphabetic knowledge, word recognition, stories, and oral language) and practices. Differences in relation to beliefs were found based on the ethnicity of teachers. African American teachers tended to believe that it was more important for the child to learn to read through teaching the alphabet (e.g., naming letters, saying their sounds), while white teachers thought it was more important for children to learn to read from teaching oral language activities (e.g., answering questions about a story or telling a story from a drawing). On the other hand, they found significant differences depending on the academic training received, so teachers with a higher academic level believed that teaching of oral language was more important, while teachers with lower academic levels did not share this belief.

Also, the report presented by the Teaching and Learning International Survey (TALIS) (OCDE, 2009) provides detailed information on the development of variables involved in the teaching and learning process. This report analyzed the beliefs of secondary school teachers in several countries. Their results indicated that most countries (Northeastern Europe, Scandinavia, Australia, and Korea) showed constructivist positions (*p* < 0.05). Humanities teachers presented more structured beliefs and were little oriented toward students (*p* < 0.05), also with differences depending on teaching experience, so the teachers with more years of experience thought and performed more structured practices (*p* < 0.05). The analyses also revealed a positive correlation between constructivist beliefs and practices in teachers from different countries (*p* < 0.05), except in Korea, where a weak relationship was found between beliefs and practices with a direct style. Finally, they found that positioning depended largely on the quality of the learning environment and job satisfaction (*p* < 0.05). In subsequent reports (OCDE, 2013), an average 95% of OECD teachers stated that they agree with constructivist practices.

Other lines of research have not found a bidirectional relationship between the teachers’ thinking and their action in the classroom. An example is the study carried out by Miglis et al. (2014) with 90 Norwegian teachers. They used a 130-item questionnaire to measure beliefs (e.g., their role as teachers, the role of teachers in teaching reading, consistency with current research about the importance of early literacy) and teaching practices (e.g., books, book contents, alphabetic knowledge, phonological awareness, and reading and writing). They found that teachers reported moderately positive beliefs about their role as a teacher in their students’ reading success, and they “agreed” with the idea that research has found that early literacy is necessary. These beliefs were not related to their practices, since the time devoted to this type of instruction was minimal. However, they discovered that the most widely used practice was “shared reading and reading aloud for 10 min a day” (29.3%). There are numerous studies that have not found a relationship between these two variables (Wilcox-Herzog, 2001). Thus, for example, through two teachers’ collaboration, Pérez-Peitx (2013) was able to observe classroom practices and analyze interviews. Their results also indicated that there was no relationship between these two variables. Along the same lines, another recent study (Utami et al., 2019) based on socio-cognitive theory studied teacher beliefs and practices in reading comprehension tasks. They found that the practices were not always consistent with their beliefs.

To our knowledge, there is no research assessing the profile of the teacher and teaching practices, in relation to all the theoretical principles that govern the teaching and learning processes of reading (i.e., innatist, maturationist, corrective, repetition, sociocultural, constructivist, psycholinguistic approaches).

The objective of this study is to find out whether or not there is a relationship between the beliefs, practices, and discourse used in teaching reading in the classroom, in order to propose more effective teaching strategies.

## Materials and Methods

The study was carried out from a mixed methods perspective, integrating qualitative and quantitative sources of information through “merge” (Creswell and Plano-Clark, 2007). The proposed design was triangulation (Morse, 2003; Creswell and Plano-Clark, 2007; Tashakkori and Teddlie, 2010; Anguera et al., 2012, 2018; Creswell, 2014), which was found suitable for the aims. A direct observation of teaching reading practices was carried out. The observational study was configured based on three criteria: study’s units, temporality, and dimensionality (Anguera et al., 2011). The observational design can be classified as Nomothetic/Follow-up/Multidimensional (N/F/M) (Sánchez-Algarra and Anguera, 2013; Portell et al., 2015). Frequency was analyzed. In order to analyze the relationship between teacher’s beliefs, practices, and discourse, a Pearson’s correlation was carried out.

### Participants

Six teachers with an age between 25 and 50 years participated. The teachers’ years of experience ranged from 10 to 35 years. They belonged to different Infant and Primary Education units on the island of Tenerife (Canary Islands, Spain). The selection criteria were based mainly on the fact that the staff member taught the subject Spanish Language and Literature, devoting an average time period of 1 h a day to the teaching of reading.

### Materials

To carry out this study, three fundamental tools were used: a questionnaire to know the teachers’ beliefs, an observation tool to analyze their practices, and a semi-structured interview to analyze the teachers’ speech about teaching and learning to read.

– *Questionnaire on Beliefs about Learning and Teaching Reading*, composed of 60 items (see Suárez et al., 2013; Jiménez et al., 2014, 2015) corresponding to the basic postulates of each learning theory: innatist, maturationist, sociocultural, constructivist, corrective, repetition, and psycholinguistic (see for review Tracey and Mandel, 2012). Teachers had to respond according to their degree of agreement or disagreement using a Likert scale of 0–10, where 0 means strongly disagree, and 10, strongly agree. Cronbach’s Alpha was 0.88.

*Observation Tool on Reading Teaching Practices.* This tool used here was developed by Suárez et al. (2018) and combines a field format and systems of categories. This consists of 14 criteria—alphabetic knowledge, phonological awareness, use of teaching resources, prior knowledge of children, reinforcement, feedback, modeling, direct instruction, guided oral instruction, extracurricular tasks, reading and writing, psychomotor skills, functional reading skills, and vocabulary—and 77 categories on practices in teaching reading. For the measurement plan, the results showed that the absolute and relative generalizability measures were acceptable (at 0.970 and 0.989) at 30 sessions and that 40 sessions were needed to reach 0.977 and 0.992, respectively. For the generalizability indexes to measure inter- and intraobserver reliability, a four-faceted SRC/O (Session, Criterion, Category/Observer) design was used, and analysis showed the greatest percentage of variability to be related to the Criterion facet (33%), while the Observer facet showed no variability at all. The absolute generalizability coefficient was 0.999, and the relative coefficient was also 0.999. With respect to the intra-rater reliability, using a four-faceted SRC/M (Session, Criterion, Category/Moment) design, analysis showed that 32% of variability corresponded to the Session facet and 33% corresponded to Criterion, while Moment showed no variability. The absolute and relative generalizability coefficients obtained for Observer 1 were both 0.999. The absolute and relative coefficients for Observer 2 were both 0.997, facet showed no variability at all. The absolute generalizability validity using a two-faceted model [Observation (2) and Criterion (74)] showed a value of 0.000 (absolute and relative validity).

– Four digital video cameras and Match Vision 3.0 software (Perea et al., 2006) were used for the sessions to record teaching practices. Data quality was analyzed using the Generalizability Study (GT) version 2.0.E program (Ysewijn, 1996) and the SAS 9.1 statistical package. Teacher discourse was analyzed using Atlas.ti 6.0 (Friese, 2011).
– *Structured Teacher Interview on Teaching Practices*. We adapted the interview on teaching perspectives elaborated by Clark and Yinger (1979), composed of 28 questions on aspects related to teaching and learning: general questions about teaching, daily classes, teaching and learning, curriculum, time, and teachers’ “philosophy.” Changes were included in the nomenclature of the subjects of the curriculum and in the section on teacher philosophy (F), where the questions were guided toward the field of reading (see Table 1).

**TABLE 1:** Interview adapted from Clark and Yinger (1979).

**General teaching issues**
1. When did you start teaching? What levels have you taught? How many years in each level?
2. How would you describe the current situation of your teaching? How long have you been teaching at this level?
3. How is your class organized today? Is there another form of organization?
4. How big is your school? Number of students? Teachers? Classrooms?
5. How is the teaching in this school? How is it similar to other places where you have previously taught?
6. What are the school’s surroundings like? Are parents and the community involved in the school?
7. Does the director impose your way of teaching? And inspections? (If yes, indicate how.)
**Everyday class**
1. What media do you consider important as a teacher? For example, equipment, space,. Do you have them in the classroom?
2. How many students do you currently have? How would you describe them as a group? How different are they from other years? What would an ideal group be like? How would you teach that group?
3. How were students assigned to your class? Did you have anything to do with that decision?
4. Do you have other people such as teaching assistants, helpers, parent volunteers, or subject specialists who help you in your class? When and for what type of activities?
**Teaching and learning**
1. How would you describe your teaching style? To what extent would you change if you had 10 students less? What if you had 10 more students?
2. In which subjects do you feel more prepared or trained? Which cause you the biggest problems? Are these the ones you enjoy more or less? To give instruction in reading, what level or year would you prefer? Why?
3. When you think about what you are going to teach and how you are going to teach it, what characteristics of your students do you have in mind? How do you notice that your performance has been improving, or getting worse? How do you think your students really learn? Do you think it is important to remedy bad learning? What could be done? If yes, how? If no, why? Do you think students with low ability should be taught in the same class?
4. How do you know that your teaching has been successful?
5. Teachers often tell me that they have enjoyed their day. Could you tell me what a good day is for you? When does it happen?
6. What has been your greatest reward in teaching your current group? Your greatest frustration?
7. I know that it is not easy to clarify it, but could you try to explain to me what you are trying to achieve most earnestly as a teacher? What do you try to achieve above all? Interview adapted from Clark and Yinger (1979).
**Curriculum**
1. What three things do you think are the most important in elementary or preschool education? What do you do to achieve them? Who decides on the content you have to teach? How do you decide your choice? What influence do you have on what you have to teach in your class? If there are strict guidelines, to what extent do you feel free to deviate from syllabus/curriculum guidelines?
2. What kinds of curriculum materials are available in the school? And in the area? What texts do you usually use? (Author/s and publisher.) Do you consider it satisfactory? If so, for what reasons? Do you group students together to learn? What criteria do you use to group them? (Tests, information from other teachers, tests, other interactions, etc.) Can you group them from more to less skilled? What kind of evaluation do you usually use? What information do you provide? When you finish the year, do you expect more or less distance between the students in your class?
**Time**
1. If you were paid five more hours a week (to devote to your work), which of the following activities would you choose to cover that extra time?
Pedagogical Renewal Collective
Personal preparation
Public relations
Teaching in class
Talking to parents
Tutorials
_________________________ (specify others if appropriate)
2. Of the following subjects (areas), which do you give the most emphasis to? Language, Mathematics, Natural Sciences, Social Sciences, Crafts.
3. If you had two more hours a week to devote to teaching, how would you distribute them taking into account the following subjects?
4. Do you have a fixed weekly schedule that you try to follow?
5. Could you describe a typical day?
**Teacher philosophy**
1. Which do you think have been most crucial in your training as a teacher and have influenced your opinions about the teaching of reading (public examinations, teachers, books, other colleagues, the experience of teaching)?
2. Reviewing the development of your notions about reading, do you think that your notions have changed from the time you were a student until now? (If so, could you specify the time and experiences that have produced these changes?)
3. Could you briefly outline your concepts of what a primary/elementary/nursery school teacher should be?

– For the interviews, a video camera and two Panasonic recorders, model RR-US455 (with 66 h of recording capacity), were used to ensure safe information storage.
– To transcribe information, the program Naturally Dragon Speaking (Baker, 1975), version 12 was employed, and Atlas.ti, version 6, for information analysis (Friese, 2011).

### Procedure

Before the recordings were made, authorization was obtained from both the teachers and the pupils’ parents. All participants provided written informed consent prior to their participation. Likewise, a schedule was agreed on for when the study would be carried out. On the day indicated, the belief questionnaire was applied to the participating teachers, their doubts in this regard were clarified, and approximately an hour was spent to complete it. Seven recording sessions per teacher (twice a week for 1 h each day) led to total of 42 h of recording (see Suárez et al., 2018). The interviews were held with the participating teachers and recorded in classrooms devoid of noise. Cameras were located in front of each teacher, and the furniture was arranged in an interview layout. The interviews of the six teachers were recorded, each lasting approximately 1 h. The audio was later transferred to the computer for the literal transcription of the interviews. Subsequently, the available information was collated and all the material subject to data processing organized. To conclude this phase, each interview was reviewed to gain an overall impression of the information provided by each teacher.

In the next phase, the document was segmented and coded through the Atlas.ti 6.0 program. The data were processed using the thematic analysis technique, according to the proposal of Braun and Clarke (2006). Initially, the hermeneutic units were defined according to the interview questions, taking into account the theories about learning to read. Subsequently, the primary documents were worked on and information segmented. In this case, we focused on words as well as phrases/sentences and texts. The relevant information was then selected, and these units were encoded. Later, we established code families composed of the different variables affecting teaching and its context. Teachers’ opinions about learning to read were categorized. The code families structured the relationship between the previously identified categories and theories on the learning of reading (e.g., innatist, maturationist, sociocultural, constructivist, corrective, repetitive, and psycholinguistic).

## Results

In order to classify each teacher according to his/her attributional profile, factor scores for each theoretical approach defined the teachers’ beliefs according to the percentiles (see Table 2).

**TABLE 2:** Teachers’ profiles in each theory in percentiles.

	**Teacher**	**F.**	**M.**	**C.**	**M.C.**	**S.**	**I.**
Theory	Sociocultural	30	70	75	5	75	5
	Maturationist	5	75	5	10	70	5
	Corrective	75	15	5	65	75	25
	Repetition	15	60	5	10	25	25
	Innatist	75	20	20	25	75	75
	Constructivism	35	30	25	25	65	45
	Psycholinguistic	35	50	55	75	40	15

**FIGURE 1:**
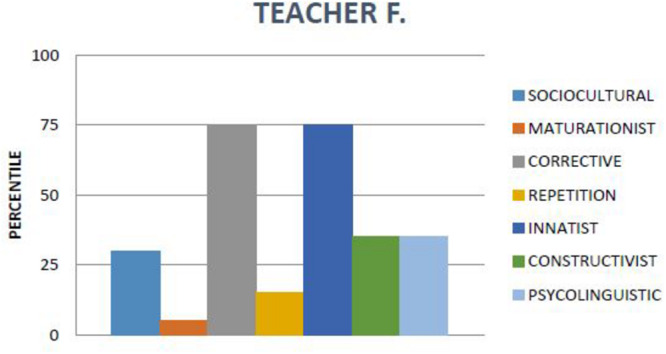
Example teacher F. profile.

To determine which theory should be attributed most to each teacher, the score was set around the percentile ≥75, and to determine which theories fitted less, around percentile ≥50 (see Figure 1).

Although all teachers were characterized by a predominant attributional profile that defined their particular beliefs, we found that their reading teaching behavior could also be attributed to any of the other theories to a lesser extent (see Table 3).

**TABLE 3:** Summary of teachers’ profiles.

**Theoretical profile**	**Teacher**
Corrective–innatist	F.
Maturationist–sociocultural	M.
Repetition	
Psycholinguistic	
Sociocultural	C.
Psycholinguistic	
Psycholinguistic	M.C.
Corrective	
Corrective/innatist/sociocultural	S.
Maturationist	
Constructivism	
Innatist	I.
Constructivism	

Regarding teaching reading practices, it was found that the most used was feedback (praising or correcting the student), followed by the use of teaching resources (e.g., stories, songs, or poetry), direct instruction (e.g., individual–group reading, aloud or silent, with or without intonation, and fluency) and functional knowledge of reading (e.g., summary, questions, comprehension exercises). To a lesser extent, they used literacy activities, reinforcement through praise (e.g., tangible or verbal), reading and writing, and work on alphabetic knowledge.

**FIGURE 2:**
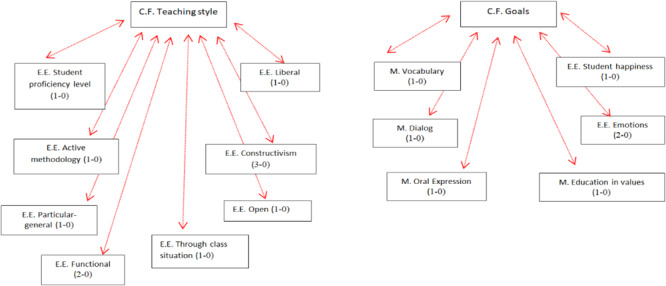
Teacher F. Network summarizing key concepts associated with the teaching process.

The latter strategy indicated that teachers mostly referred to constructivist theory, except teacher M.C., who chose to position herself in psycholinguistic theory. Similarly, teacher F. emphasized that students should build their learning and that teachers should function as a guide. To a lesser extent, she commented on aspects of the maturation and behaviorist theory (see Figure 2). Teacher M. also focused on the foundations of constructivism (e.g., prior knowledge, children discover their learning). She also talked about the importance of psychomotor skills, correctness in reading, as well as the involvement of parents. Teacher C. commented that students learn through construction and must discover reading autonomously through the support offered by the teacher. She also emphasized the role that parents play in reading, the importance of resources, oral language work, phonological awareness, as well as maturity in the development of reading. Teacher M.C. placed greater emphasis on the development of phonological awareness and oral language to teach reading. However, teacher S. focused more on student autonomy in the learning process and to a lesser extent on oral language, use of resources, and correction during reading (feedback). Teacher I. focused mostly on the construction of learning and less so on the role of oral language and the use of resources (library).

Subsequently, the information was triangulated after analyzing the beliefs, practices, and discourse of the teachers. For this, several researchers who are experts in the learning and teaching of reading skills agreed on the following relationship, in accordance with the basic postulates of each of the theories considered (see Table 4).

**TABLE 4:** Triangulation between theoretical profile, teaching practices, and teacher discourse.

**Theory**	**Teaching practices**	**Speech**		
		**Teaching**	**Learning**	**Context**
Sociocultural	Teaching resources		Sociocultural	Neighborhood Available resources
	Homework			School requirements
				Parents/teacher support
Maturationist	Psychomotor skills		Maturationist	*Not contemplated*
Corrective	Feedback			*Not contemplated*
Repetitive	Reinforcement	Evaluation through observation	Repetitive	*Not contemplated*
	Modeling			
	Direct instruction			
	Guided oral Instruction			
Innatist	Practices were not observed	Programming	Innatist	*Not contemplated*
		Individual/group/pair		
		organization		
Constructivism	Teaching resources	Modeling	Constructivist	*Not contemplated*
	Previous knowledge	Self-appraisal		
	Reading and writing	Construction knowledge		
		Previous knowledge		
		Situations that arise in the classroom		
		Autonomy		
		Liberal		
		Phonological		
Psycholinguistic	Phonemic awareness	Syllabic	Psycholinguistic	*Not contemplated*
	Alphabetic knowledge	General–specific		
	Vocabulary	Specific–general		
	Fluency	Oral expression		

Then the teachers’ scores were compared in relation to their beliefs, teaching practices (in terms of frequency), as well as teacher discourse, previously analyzed through its categorization into teaching–learning processes and their context (see Table 5). Finally, the results were interpreted according to Pearson’s correlation analysis. The results showed a high correlation (*r* = 0.72, *p* < 0.05) in teacher F. and in teacher I. (*r* = 0.71, *p* < 0.05) and a negative and high correlation in teacher M. (*r* = −0.81, *p* < 0.05) between beliefs and practices. Moreover, they showed a moderate correlation in teacher C. (*r* = 0.52) and in teacher M. (*r* = 0.45) between beliefs and discourse. Finally, the results showed a negative and high correlation in teacher I. (*r* = −0.74, *p* < 0.05) and in teacher M.C. (*r* = −0.76, *p* < 0.05) between practices and discourse.

**TABLE 5:** Percentages of teachers’ beliefs, reading practices, and discourse.

	**Teachers’ beliefs %**						**Reading practices %**						**Teachers’ discourse %**					
**Theory**	**F.**	**M.**	**C.**	**M.C.**	**S.**	**I.**	**F.**	**M.**	**C.**	**M.C.**	**S.**	**I.**	**F.**	**M.**	**C.**	**M.C.**	**S.**	**I.**

Sociocultural	11.1	22	39.6	2.4	17.6	2.6	5.3	5.7	4.4	4.4	4.4	10.6	**	14.3	33.3	3.7	21.7	10
Maturationist	1.8	23.4	2.6	4.6	16.6	2.6	2.2	0.6	0.6	0.3	1.4	0.2	11.1	21.4	22.3	**	4.3	**
Corrective	27.8	4.8	2.6	30.2	17.6	12.8	29.2	32.6	50.8	37.6	35.1	23	11.1	14.3	**	14.8	13	**
Repetition	5.6	18.7	2.6	4.7	5.9	12.8	23.5	25.2	16.9	19.6	36	25.3	**	**	**	7.4	**	**
Innatist	27.7	6.2	10.6	11.6	17.6	38.5	*	*	*	*	*	*	**	**	**	**	**	**
Constructivism	13	9.4	13.1	11.6	15.3	23	19.9	19.1	10.9	13	9.6	19.3	77.8	50	33.3	14.8	47.9	70
Psycholinguistic	13	15.6	28.9	34.9	9.4	7.7	16	16.6	12	4.1	8.5	3.7	**	**	11.1	59.3	13	20

*Practices not observed. **Opinion about different theories not contemplated in teachers’ discourse.

Teacher F. showed links between his theoretical profile and his practices. A relationship between corrective beliefs (27.8%) and practices (29.2%) was found. On the other hand, we observed that in his practices, he used activities associated with other theories: repetition (23.5%), constructivism (19.9%), and psycholinguistic (16%). This also happened when he thought about how children learn to read, since he considered that the construction of learning (77.8%), maturation (11.1%), and providing feedback (11.1%) were fundamental. Other discourse makers, teacher M. did not show a link between her sociocultural (22%) and maturationist (23.4%) theoretical profile and her practices (5.7% and 0.6%). However, the results indicated that her maturationist (23.4%), sociocultural (22%) beliefs were related only to her discourse. So, she thought that the use of psychomotor skills (21.4%), teaching resources such as stories, stories, poems, and texts (14.3%), and teaching previous knowledge (50%) were important. However, practices based on other currents were observed: corrective reading (32.6%) and repeated reading (25.2%), as well as constructivism (19.1%), such as working previous knowledge or reading and writing and psycholinguistic skills (16.6%) [e.g., alphabetic knowledge: teaching letter names and sounds, rules with support rhymes, etc.; phonological awareness: stimulating children to become aware of letter sounds, saying words that begin with a certain sound, separating words into syllables, playing the game *veo-veo* (I spy.); vocabulary: teaching the meaning of words]. During the interview, opinions related to other theories were also found (i.e., corrective).

As for teacher C., there was a bidirectional relationship between her sociocultural theoretical profile (39.6%) (e.g., use of teaching resources such as stories, songs, writings from different sources, etc.) and her discourse (33.3%). Also, it was found that her psycholinguistic profile (28.9%) was related to her discourse (11.1%) (e.g., oral language or phonological awareness). However, the results indicated that this teacher carried out other practices not related to her theoretical beliefs, such as: feedback (50.8%) and repetition (16.9%). The same occurred with her discourse; she thought that maturation was also important (22.3%).

Regarding teacher M.C., a negative relationship was found between her psycholinguistic discourse (59.3%) and her teaching practices (4.1%). The same happened with her corrective practices (37.6%) and her discourse (14.8%) (e.g., correct when the child is wrong, point out, provide examples, deny). However, when we analyzed her practices, we found activities justified by other theories, such as functional knowledge of reading or use of teaching resources (13%) or repetition (19.6%) and constructivism (13%) (e.g., previous reading and writing, and likewise when we asked her opinion about how children learn to read (e.g., constructivism).

Regarding teacher S., she showed a corrective (17.6%), innatist (17.6%), sociocultural (17.6%), maturationist (16.6%), and constructivism (15.3%) profile. Then, she carried out corrective (35.1%) practices (e.g., feedback, direct instruction). During her discourse, opinions were also found that were constructivist (47.9%) and psycholinguistic (20%). Nevertheless, repetition practices (36%) were observed that had nothing to do with her expressed beliefs.

A relationship was found between the constructivism profile (23%) of teacher I. and her practices (19.3%). Then the result showed a relationship between corrective (12.6%) and repetitive (12.6%) beliefs and practices. Furthermore, this teacher used other practices unrelated to any of her attributed beliefs, such as: sociocultural (10.6%). No relationship between corrective (23%) and repetition (25.3%) practices and discourse were found. In the same way, she referred to the implication of other (e.g., sociocultural and psycholinguistic) theories in infant readers’ learning. The innatist profile of teacher I. was not related to her practices or discourse.

## Discussion

The results of the present study are congruent with previous study results that showed that teachers hold eclectic positions (Clemente, 2008; Jiménez and O’Shanahan, 2008; Clemente et al., 2010; Rodríguez and Clemente, 2013). Other research has shown quite different results, from studies finding a relationship between beliefs and teaching practices in reading learning (Cunningham and Zibulsky, 2009; Tolchinsky and Ríos, 2009; Rapoport et al., 2016) to studies which indicated a moderate correlation (Baumann et al., 1998). On the opposite side, other authors found no such relationship (Pérez-Peitx, 2013; Miglis et al., 2014; Enyew and Melesse, 2018; Utami et al., 2019).

The data extracted from the belief questionnaires have been complemented with the analysis of teaching practices and each teacher’s interviews, which allowed us to provide additional information (Castañer et al., 2013). In our case, the interview helped us complete the teacher’s profile. We found that the teaching and learning processes are mediated by multiple contextual variables that were not identified by the questionnaire or recorded observations.

Analysis of the practices allowed us to identify not only what activities the teachers performed in their real teaching context but also how their sequence of instruction was oriented in all cases toward the use of their own multiple resources, applying other theories. The relationship found between some beliefs and practices in this study suggests that if teachers are aware of their own beliefs, the repertoire of teaching practices can be increased (Tracey and Mandel, 2012), causing changes in decision making in the classroom and in teaching and evaluation strategies. In addition, as all teachers used many activities characteristic of other theories they did not explicitly hold, we focused on the opposite process, modifying their practices to cause a change in their beliefs (Fazio, 2003), since these are permeable mental structures that can be modified (Thompson, 1992). But how can we achieve this? Some studies confirm that people form their implicit theories through the knowledge they acquire (Suárez and Jiménez, 2014).

The first step is to achieve the teacher’s predisposition to change, always through invitation (Baena, 2000), by encouraging reflection. To do this, they should become aware how their own beliefs are involved in their teaching practice and how they influence student performance. In addition, the false myths about learning to read and teaching practices should be recognized, as prescribed by the National Reading Panel [NRP] (2000). The question remains whether teachers have received training based on the latest advances in scientific research on the teaching of reading, in order to provide young students (who may or may not have difficulties) with the tools necessary for their learning to proceed optimally.

Online training offers teachers the opportunity to recycle their knowledge (Costi et al., 2005; Jiménez, 2015; Jiménez et al., 2015; Jiménez and O’Shanahan, 2016), which generates an important pillar supporting success, integration, and sustainability in education (Haydon and Barton, 2007; Somekh, 2008). It is also an alternative solution to the lack of time and difficulties in reconciling work and family life. It has been found that experience with these resources plays a fundamental role, since it favors a positive attitude of teachers and also confidence in the use of these tools for education (BECTA, 2009). Joshi et al. (2009) found that the training teachers receive is inadequate because textbooks and courses in education reflect superstitions, anecdotes, and beliefs that are not based on scientific evidence. Research has also found that teachers do not properly use the practices that are based on scientific evidence (Moats, 2009). If the learning environment is effective, it can even happen that only a small percentage of students present difficulties in learning to read (Cunningham and Zibulsky, 2009).

The updating of knowledge according to research conclusions is proposed as an alternative for teachers who specialize in teaching reading, since teaching quality is one of the main factors determining the academic success of students (European Council, 2008). For teachers to learn good practices, it is important that they have the following knowledge at their disposal: (1) fundamental research and theories about the development of language and reading; (2) strategies for use in the classroom to teach word recognition, vocabulary, text comprehension, and fluency; (3) tools to work on reading and writing at the same time; (4) the best strategies to teach reading and the materials to use; (5) different techniques for student evaluation; (6) how to maintain a good balance between theory, practice, and information technologies; (7) knowledge of dyslexia and other learning disorders (IRA, 2007); and (8) how to interpret and administer assessment tests to plan teaching (IDA, 2010). In addition, they must learn to ask more complex questions to help students make inferences and more elaborate reflections, as well as work with students’ prior knowledge (RAND, 2002). However, the teacher alone should not be responsible for this process, because we have confirmed that in the teaching environment, there are other strong factors such as society or culture (Quintana, 2001). The challenge now consists of achieving a change in the ways of thinking of those responsible for educational administration. The necessary means should also be provided to facilitate refresher courses and ongoing e-learning for teachers, with training programs that include content based on scientific evidence. One limitation is that the study consisted of six teachers and is not generalizable to a greater audience.

## Conclusion

In general terms, we can conclude that the relationship between beliefs, practices, and discourse varies according to certain nuances. Thus, of the two beliefs attributed to teacher F., only one (corrective) was related to his form of instruction and his opinion. Among the four beliefs attributed to teacher M. (sociocultural, maturationist, repetition, and psycholinguistic), a relationship was found only between her maturationist and sociocultural profile and her discourse. Both beliefs attributed to teacher C. (sociocultural and psycholinguistic) were related to the discourse content. Of the two beliefs attributed to teacher M.C. (corrective and psycholinguistic), neither of them was related to her actions and reflections. Among the five beliefs attributed to teacher S. (sociocultural, innatist, corrective, maturationist, and constructivist) only two (corrective and sociocultural) were related to her active practices and discourse comments. Finally, of the two beliefs of teacher I. (innatist and constructivist), only constructivism was related to her practices or her opinion.

Although it is true that a relationship was found in all the teachers between some of their beliefs, practices, and discourse, as revealed in their discursive talks, all the teachers thought that learning to read depended on factors underlying other theories not related to their attributional profile. Therefore, despite attributing to them certain beliefs when they teach children to read and when they think of learning to read, it can be concluded that all teachers maintain an eclectic approach.

## Data Availability Statement

All datasets generated for this study are included in the article/supplementary material.

## Ethics Statement

Ethical review and approval was not required for the study on human participants in accordance with the local legislation and institutional requirements. Written informed consent to participate in this study was provided by the participants’ legal guardian/next of kin.

## Author Contributions

NS: this author’s grant was used to run the project Integrando creencias y prácticas de enseñanza de la lectura (Integrating beliefs and practices about teaching reading), ref: PSI2009-11662. She participated actively in the research, analyzed the teaching practices and discourse, and was responsible for the literature review and drafting of this manuscript. JJ: supervised the project and the preparation of the study, offered theoretical guidance, and was responsible for reviewing the manuscript. CS: supervised the design and preparation of the study, offered guidance on methodology, and helped review the manuscript. All authors approved the final version of this article.

## Conflict of Interest

The authors declare that the research was conducted in the absence of any commercial or financial relationships that could be construed as a potential conflict of interest.

## Figures and Tables

**Table:** 

**Concepts**	**Principal postulate**
Repetition theory	The importance of repetition, reinforcement, modeling direct instruction, and guided oral Instruction to teaching to read.
Corrective theory	The teacher gives feedback (e.g., correct reading, line jump, incorrect pronunciation, report errors, make questions) to the students to
	read well.
Constructivist theory	The students are the builders of their own learning, meaningful learning prevails, and the teacher acts as support.
Maturationist theory	For reading, children need to develop space–time orientation. Not all of them learn at the same time; it depends on their stage and rate
	of development.
Innatist theory	Children can read at early ages.
Sociocultural theory	The role of the family, school, instruction and characteristics of the teacher, society, regulatory laws, and the curriculum of each autonomous
	region (state etc.) play a crucial role in reader learning.
Psycholinguistic theory	The importance of phonological awareness in the teaching of reading, as well as other cognitive processes: lexical, syntactic, semantic.

## References

[B1] Anguera M. T. Blanco A. Hernández A. Losada J. L. (2011). Diseños observacionales, ajuste y aplicación en psicología del deporte. [Observational designs, adjustment and application in Sports Psychology]. *Cuad. Psych. Deport.* 11 63–76.

[B2] Anguera M. T. Camerino O. Castañer M. (2012). “Mixed methods procedures and designs for research on sport, physical education and dance,” in *Mixed Methods Research in the Movement Sciences: Case studies in sport, physical education and dance*, eds Camerino O. Castañer M. Anguera M. T. (Abingdon: Routledge), 3–27. 10.4324/9780203132326

[B3] Anguera M. T. M. Chacón-Moscoso S. Sanduvete-Chaves S. (2018). Indirect observation in everyday contexts: concepts and methodological guidelines within a mixed methods framework. *Front. Psychol.* 9:13. 10.3389/fpsyg.2018.00013 29441028 PMC5797623

[B4] Baccus A. A. (2004). *Urban Fourth and Fifth Grade Teachers’ Reading Attitudes and Efficacy Beliefs: Relationships to Reading Instruction and to Students’ Attitudes and Efficacy Beliefs.* Ph.D. thesis, University of Maryland, College Park, MD.

[B5] Baena D. (2000). Pensamiento y acción en la enseñanza de las ciencias [Thought and action in science education]. *Invest. Didác.* 18 217–226.

[B6] Baker J. (1975). The DRAGON System - An Overview. *IEEE Trans. Acoust. Speech Sign. Process.* 23 24–29. 10.1109/TASSP.1975.1162650

[B7] Barragán C. Medina M. M. (2008). Las prácticas de la lectura y escritura en Educación Infantil [Reading and writing practices in Elementary Education]. *Rev. de Educ.* 10 149–165.

[B8] Barrot J. S. (2015). A socio-cognitive-transformative instructional materials design model for second language (L2) pedagogy in the Asia Pacific: development and validation. *Asia Pac. Educ. Res.* 24 283–297. 10.1007/s40299-014-0179-0

[B9] Baumann J. Hoffman J. Moon J. Duffy-Hester A. (1998). Where are teachers’ voices in the phonics/whole language debate? Results from a survey of U. S. elementary classroom teachers. *Read. Teach.* 51 636–650.

[B10] BECTA (2009). *The Becta Review 2009: Evidence on The Progress of ICT in Education.* Coventry: BECTA.

[B11] Berthelsen D. Brownlee J. (2007). Working with toddlers in childcare: practitioners’ beliefs about their role. *E. Child. Res. Quart.* 22 347–362. 10.1016/j.ecresq.2006.12.002

[B12] Block J. H. Hazelin K. (1995). “The teacher’s beliefs and belief systems,” in *International Encyclopedia of Teaching and Teacher Education*, 2nd Edn, ed. Anderson L. W. (New York, NY: Pergamon), 25–28.

[B13] Braun V. Clarke V. (2006). Using thematic analysis in psychology. *Qualit. Res. Psychol.* 3 77–101. 10.1191/1478088706qp063oa 32100154

[B14] Castañer M. Camerino O. Anguera M. T. (2013). Métodos mixtos en la investigación de las ciencias de la actividad física y el deporte [Mixed methods in physical activity and sports science research]. *Ap. Edu. Fís. i Esp.* 112 31–36. 10.5672/apunts.2014-0983.es.(2013/2).112.01

[B15] Center for the Improvement of Early Reading Achievement [CIERA] (2001). *Put. (Reading)First: The Research Building Blocks for Teaching Children to Read.* Washington DC: National Institute for Literacy.

[B16] Clark C. Yinger R. (1979). Research on teacher planning: a progress report. *J. Curric. Stud.* 11 175–177. 10.1080/0022027790110209

[B17] Clemente L. Rodríguez E. Sánchez M. C. (2010). Enfoques teóricos y prácticas docentes en la enseñanza inicial de la lengua escrita [Theoretical approaches and teaching practices in the initial teaching on written language]. *Infan. y Aprend.* 22 313–328. 10.1174/113564010804932175 25131292

[B18] Clemente M. (2008). La complejidad de las relaciones teoría-práctica en educación [The complexity of theory-practice relationships in education]. *Rev. Teo. de la Educ.* 19 25–46.

[B19] Costi L. Passerino L. Carnero M. Geller M. (2005). *Programa de Formación de Profesores a Distancia y En Servicio. Visando la Inclusión Digital/Social. PROISNEP [Training Program for Distance and in-Service Teachers. Seeking Digital/Social Inclusion].* Avalaible online at: http://capacidad.es/ciiee07/Brasil.pdf (accessed May 5, 2019).

[B20] Creswell J. W. (2014). *A Concise Introduction to Mixed Methods Research.* Thousand Oaks, CA: SAGE Publications.

[B21] Creswell J. W. Plano-Clark V. L. (2007). *Designing and Conducting Mixed Methods Research.* Thousand Oaks, CA: Sage.

[B22] Cunningham A. E. Zibulsky J. (2009). Perspectives on teachers’ disciplinary knowledge of reading processes, development, and pedagogy. *Read. Writ.* 22 375–378. 10.1007/s11145-009-9161-2

[B23] Enyew C. Melesse S. (2018). Nexus between beliefs college english instructors’ held about teaching reading strategies and their classroom practice. *Res. Pedag.* 8 121–131. 10.17810/2015.78

[B24] European Council (2008). *Presidency Conclusions of the Brussels European Council, 13-14 March, 2008. 7652/1/08 REV 1.20 May.* Brussels: European Council.

[B25] Fazio M. (2003). Constructive comprehension and metacognitive strategy reading instruction in a field-based teacher education program: effecting change in preservice and in-service teachers, participant one. *Year. Coll. Read. Asstn.* 25 23–45.

[B26] Friese S. (2011). *Atlas. Ti 6. User Guide and Reference.* Berlin: Scientific Software Development GmbH.

[B27] Haydon T. Barton R. (2007). First do no harm: developing teachers’ ability to use ICT in subject teaching: some lessons from the UK. *Br. J. Educ. Tech.* 38 365–368. 10.1111/j.1467-8535.2006.00639.x

[B28] IDA (2010). *Knowledge and Practice Standards for Reading Teachers.* Baltimore: International Dyslexia Association Individuals with Disabilities Education.

[B29] IRA (2007). *Teaching Reading Well: A Synthesis of the International Reading Association’s Research on Teacher Preparation for Reading Instruction.* Newark, DE: IRA.

[B30] Jiménez J. E. (2015). “The letra program: a web-based tutorial model for preparing teachers to improve reading in early grades,” in *Advances in Reading Intervention: Research to Practice to Research*, eds McArdle P. Connor C. (Baltimore, MD: Brookes Publishing Co), 181–195.

[B31] Jiménez J. E. O’Shanahan I. (2008). Enseñanza de la lectura: de la teoría y la investigación a la práctica educativa [Teaching reading: from theory and research to educational practice]. *Rev. Iber. Educ.* 45 5–25. 10.35362/rie4552032

[B32] Jiménez J. E. O’Shanahan I. (2016). Effects of web-based training on Spanish pre-service and in-service teacher knowledge and implicit beliefs on learning to read. *Teach. Teach. Educ.* 55 175–187. 10.1016/j.tate.2016.01.006

[B33] Jiménez J. E. Rodríguez C. Suárez N. O’Shanahan I. (2014). Coinciden nuestras ideas con lo que dicen las teorías científicas sobre el aprendizaje de la lectura? [Do our ideas coincide with what scientific theories say about learning to read?]. *Rev. Esp. de Pedag.* 259 395–412.

[B34] Jiménez J. E. Rodríguez C. Suárez N. O’Shanahan I. Villadiego Y. Uribe C. (2015). Teacher implicit theories of learning to read: a cross-cultural study in Iberoamerican countries. *Read. Writ.* 28:6 10.1007/s11145-015-9574-z

[B35] Joshi R. M. Binks E. Hougen M. Dahlgren M. Oker-Dean F. Smith D. (2009). Why elementary teachers might be inadequately prepared to teach reading? *J. Lear. Disabil.* 42 444–457. 10.1177/0022219409338736 19542350

[B36] Kuzborska I. (2011). Links between teachers’ beliefs and practices and research on reading. *Read. For. Lang.* 23 102–128.

[B37] McAlpine L. Weston C. (2002). “Reflection: issues related to improving teachers’ teaching and students’ learning,” in *Teacher Thinking, Beliefs and Knowledge in Higher Education*, eds Goodyear Hativa (Dordrecht: Kluwer), 59–78. 10.1007/978-94-010-0593-7_4

[B38] Miglis J. van Daal V. Dèar H. (2014). Emergent literacy: preschool teachers’ beliefs and practices. *J. Early Child. Liter.* 14 28–52. 10.1177/1468798413478026

[B39] Moats L. (2009). Knowledge foundations for teaching reading and spelling. *Read. Writ.* 22 379–399. 10.1007/s11145-009-9162-1

[B40] Morse J. M. (2003). “Principles of mixed methods and multimethod research design,” in *Handbook of Mixed Methods in Social and Behavioral Research*, eds Tashakkori A. Teddlie C. (Thousand Oaks, CA: Sage), 189–208.

[B41] National Reading Panel [NRP] (2000). *Teaching Children to Read: An Evidence-Based Assessment of the Scientific Research Literature on Reading and Its Implications for Reading Instruction: Reports of the Subgroups.* Bethesda, MD: National Institute of Child health and Human Development.

[B42] OCDE (2009). *TALIS. Teaching and Learning International Survey.* Madrid: Ministerio de Educación 10.1787/9789264068780-en

[B43] OCDE (2013). *TALIS. Teaching and Learning International Survey.* Madrid: Ministerio de Educación.

[B44] Perea A. Castellano J. Alday N. (2006). *MatchVision Studio Premium V.3.* Vitoria: Universidad del País Vasco.

[B45] Pérez-Peitx M. (2013). Perfils de pràctiques docents i creences de dues mestres de parvulari.[Practical profiles and teacher’s beliefs in Kindergarten]. *Bellat. J. Teach. Learn. Lang. Literat.* 6 56–71. 10.5565/rev/jtl3.473

[B46] Portell M. Anguera M. T. Chacón-Moscoso S. Sanduvet S. (2015). Guidelines for reporting evaluations based on observational methodology. *Psicoth* 27 283–289. 10.7334/psicothema2014.276 26260937

[B47] Quintana J. M. (2001). *Las creencias y la educación [Beliefs and education].* Pedagogía cosmovisual, Barcelona: Herder.

[B48] RAND (2002). *Reading for understanding: Towards an R&D program in reading comprehension.* Santa Monica, CA: RAND.

[B49] Rapoport S. Rubinsten O. Katzir T. (2016). Teachers’ beliefs and practices regarding the role of executive functions in reading and arithmetic. *Front. Psychol.* 7:1567. 10.3389/fpsyg.2016.01567 27799917 PMC5065981

[B50] Richardson V. (1996). “The role of attitudes and beliefs in learning to teach,” in *Handbook of Research on Teacher Education*, 2nd Edn, ed. Sikula J. (New York: Macmillan), 102–119.

[B51] Ríos I. Fernández P. Gallardo I. (2010). *La contribución de las prácticas de aula a los logros de aprendizaje [The contribution of classroom practices to learning achievements]. II Congrés Internacional de didactiques.* Girona: Universitat 322–329.

[B52] Rodríguez I. Clemente M. (2013). Creencias, intenciones y prácticas en la enseñanza de la lengua escrita: estudio de caso [Beliefs, intentions and practices in the teaching of written language]. *Rev. Elec. Interu. de Form. del Prof.* 16 41–54. 10.6018/reifop.16.1.179431

[B53] Sánchez-Algarra P. Anguera M. T. (2013). Qualitative/quantitative integration in the inductive observational study of interactive behaviour: impact of recording and coding predominating perspectives. *Qual. Quant.* 47 1237–1257. 10.1007/s11135-012-9764-6

[B54] Somekh B. (2008). “Factors affecting teachers’ pedagogical adoption of ICT,” in *International Handbook of Information Technology in Primary and Secondary Education*, eds Voogt J. Knezek G. (Amsterdam: Springer), 449–460. 10.1007/978-0-387-73315-9_27

[B55] Stevens L. P. (2002). Making the road by walking: the transition from content area literacy to adolescent literacy. *Read. Res. Instr.* 41 267–278. 10.1080/19388070209558370

[B56] Suárez N. Jiménez J. E. (2014). ¿Influyen los años de experiencia y la especialidad de los profesores en las teorías implícitas que se atribuyen sobre el aprendizaje de la lectura? [Do years of experience and expertise of teachers influence the implicit theories attributed to learning to read?]. *Int. J. Dev. Educ. Psychol.* 2 257–262. 10.17060/ijodaep.2014.n1.v2.438

[B57] Suárez N. Jiménez J. E. Rodríguez C. O’Shanahan J. Guzmán R. (2013). Las teorías sobre la enseñanza de la lectura desde una perspectiva sociohistórica. [Teaching Reading theories from a sociohistorical perspective]. *Rev. Psic. Educ.* 8 171–186.

[B58] Suárez N. Sánchez C. R. Jiménez J. E. Anguera M. T. (2018). Is reading instruction evidence-based? Analyzing teaching practices using T-patterns. *Front. Psychol.* 9:7. 10.3389/fpsyg.2018.00007 29449818 PMC5800299

[B59] Tashakkori A. Teddlie C. (2010). *Sage Handbook of Mixed Methods in Social & Behavioral Research.* Thousand Oaks, CA: Sage Publications 10.4135/9781506335193

[B60] Theurer J. L. (2002). The power of retrospective miscue analysis: one preservice teacher’s journey as she reconsiders the reading process. *Read. Matr.* 2:1.

[B61] Thompson A. G. (1992). “Teachers’ beliefs and conceptions: a synthesis of the research,” in *Handbook of Research in Mathematics Teaching and Learning*, ed. Grouws D. A. (New York, NY: Macmillan), 127–146.

[B62] Tolchinsky L. Ríos I. (2009). ¿Qué dicen los maestros que hacen para enseñar a leer y a escribir? [What do teachers say they do to teach reading and writing?]. *Au. de Innov. Educ.* 174 1–7.

[B63] Tracey D. Mandel L. (2012). *Lenses on Reading: An Introduction to Theories and Models.* New York: The Guilford Press.

[B64] Utami L. Nurkamto J. Suryani N. Gunarhadi (2019). Teacher’s beliefs and practices in teaching reading: a sociocognitive perspective. *Intern. J. Adv. Res.* 7 127–135. 10.21474/IJAR01/9203

[B65] Vartuli S. (2005). Beliefs: the heart of teaching. *Young Child.* 60 76–86.

[B66] Wilcox-Herzog A. (2001). Is there a link between teachers’ beliefs and behaviors? *Educ. Dev.* 13 81–106. 10.1207/s15566935eed1301_5

[B67] Ysewijn P. (1996). *GT Software for Generalizability Studies.* London: Mimeograph.

